# The relationship between loneliness and problematic social media usage in Chinese university students: a longitudinal study

**DOI:** 10.1186/s40359-023-01498-4

**Published:** 2024-01-04

**Authors:** Peibo Wu, Rong Feng, Jindan Zhang

**Affiliations:** 1 Institute of Education, Xiamen University, Xiamen, People’s Republic of China; 2 Zhong Yuan Institute of Science and Technology, Zhengzhou, People’s Republic of China; 3 Propaganda Department, Taizhou University, Taizhou, People’s Republic of China

**Keywords:** Loneliness, Problematic social media use, College students, Latent growth model, Cross-lagged model, Longitudinal study

## Abstract

**Background:**

A significant number of cross-sectional studies have explored the correlation between loneliness and problematic social media use. However, the causal relationship between these two key variables remains controversial, and the developmental relationship over time is unclear.

**Methods:**

We conducted a one-year longitudinal study with 538 Chinese college students using questionnaires and employing cross-lagged and latent growth models to investigate the causal relationship and developmental dynamics between loneliness and problematic social media use.

**Results:**

The results indicate that (a) loneliness and problematic social media use mutually and positively influence each other, establishing a bidirectional causal relationship; (b) Chinese college students experience a gradual increase in levels of loneliness and problematic social media use during their time in college; and (c) the intercept and slope of loneliness significantly and positively affect the intercept and slope of problematic social media use, and conversely, the intercept and slope of problematic social media use significantly and positively influence the intercept and slope of loneliness.

**Conclusion:**

These findings reveal the longitudinal relationship between loneliness and problematic social media use among Chinese college students and offer insights for researchers and educators to intervene in college students' loneliness and problematic social media use from a developmental perspective.

## Introduction

With the rapid development of information technology, social media on the internet has swiftly evolved into the primary platform for people’s social interaction and leisure activities. It combines features such as interpersonal communication, online gaming, information browsing, self-presentation, and multimedia entertainment. Consequently, an increasing number of individuals, particularly college students, are engaging in high-frequency and high-intensity use of social media [1]. The ever-changing forms and diverse functionalities of social media fulfill people's psychological needs for social activities but have also given rise to adverse effects. Among users, particularly college students, there is a growing phenomenon of problematic social media use, characterized by individuals’ excessive dependency on social media driven by strong motivation [2, 3]. Problematic social media use refers to the phenomenon in which individuals experience negative physical and psychological effects due to prolonged and intensive use of social media [4]. Extensive research has shown that problematic social media use not only affects the academic and personal lives of adolescents but also harms their mental and physical health [5, 6]. It leads to issues such as deteriorating academic performance, worsening interpersonal relationships [7], mental health problems such as depression and anxiety [8, 9], and impacts on memory function [10]. Considering the significant risks posed to the healthy development of college students by problematic social media use, researchers have attempted to identify factors that contribute to this phenomenon. Among various factors, the literature documents a significant positive correlation between loneliness and problematic social media use. Individuals with a stronger sense of loneliness often exhibit more severe problematic social media use [11, 12].

Loneliness is defined as a subjective perception of a misalignment between one's expectations and the actual state of social relationships [13]. While a certain degree of correlation between loneliness and problematic social media use has been established, there is still a need for further research into the relationship between these two factors. First, there is a lack of longitudinal studies that explore the mutual predictive relationship between loneliness and problematic social media use. Existing research on the predictive direction of the relationship between loneliness and problematic social media use has yielded inconsistent findings [11, 14–19]. It remains unclear whether loneliness precedes or follows problematic social media use. Second, the developmental trajectories of loneliness and problematic social media use as well as their interactions require further investigation. Some studies suggest that individuals' levels of loneliness and problematic social media use change over time [16, 20–23], implying that these two factors may interact as they evolve.

This study aimed to address these gaps by collecting three waves of longitudinal data among Chinese college students. First, we utilized a cross-lagged model to explore the causal sequence between loneliness and problematic social media use. Building upon the cross-lagged model, we further employed a latent variable growth model to investigate the developmental trajectories of loneliness and problematic social media use. From a developmental perspective, we examined the mutual influence between loneliness and problematic social media use. The two a forementioned studies provide a theoretical foundation for intervening in college students’ loneliness and problematic social media use during their developmental journey.

### The causal relations between loneliness and problematic social media use

Current research suggests a correlational relationship between loneliness and problematic social media use [11, 12], but there is some debate regarding the direction of this relationship. Three main perspectives have emerged. The first perspective posits that loneliness positively predicts problematic social media use [11, 15, 19, 24]. The second perspective suggests that problematic social media use positively predicts loneliness [14, 18]. The third perspective proposes a bidirectional relationship between loneliness and problematic social media use [16, 17].

The first perspective suggests that loneliness positively predicts problematic social media use. According to the Need-Satisfaction Theory, loneliness, as an unmet psychological need, often leads individuals to use social media as a means of compensation. During this compensatory process, the individual might increase the frequency and intensity of social media use, leading to problematic usage [25]. Loneliness is typically the unpleasant experience that arises when there are significant deficiencies in an individual's social network [26]. When individuals find it challenging to establish or maintain satisfying interpersonal relationships, it may lead to negative emotions such as anxiety and depression, accompanied by a strong desire to connect with others [27, 28]. Social media provide individuals with an expanding social network, and the anonymity of online communication makes it easier for people to engage and interact, offering companionship and a sense of belonging. Thus, lonely individuals may be drawn to certain forms of online social interactions. Research has also indicated that lonely individuals tend to prefer online social interactions on social media platforms [19] and are more inclined to seek social fulfillment online to compensate for social deficits in their offline lives [11, 29]. Studies such as the one conducted by Błachnio et al. [30] have found that highly lonely individuals spend more time and engage more frequently on mobile social networks than less lonely individuals. The stronger an individual’s feelings of loneliness are, the more likely the individual is to exhibit problematic social media use [30]. Therefore, loneliness is considered a predictive factor for problematic social media use.

The second perspective posits that problematic social media use positively predicts loneliness. Drawing from reinforcement theory, if a behavior yields positive outcomes or reduces adverse results, it is more likely to be repeated [31]. This mechanism is evident in the context of problematic social media use. Users who spend extended periods on social media platforms often sacrifice real-life relationships, opting to cultivate weak online connections with strangers [32]. While this behavior may provide temporary solace as an escape from reality in the short term, in the long run, it weakens individuals' connection to the real world, diminishing their sense of belonging in real-life interpersonal relationships [32]. Furthermore, problematic social media users often find that online interactions on networking platforms fail to offer deep interpersonal satisfaction. These superficial online interactions may exacerbate feelings of technological alienation, contributing to communication barriers [33]. These barriers not only hinder genuine social interactions but may also lead to a deeper sense of loneliness. Research indicates a positive predictive relationship between problematic social media use and loneliness [14, 34], higher levels of problematic social media use associated with stronger feelings of loneliness.

The third perspective proposes a bidirectional relationship between loneliness and problematic social media use. As emphasized by the Person-Affect-Cognition-execution (I-PACE) model, which explains the formation and persistence of addictive behaviors, internal factors such as cognition and affect can trigger addictive behaviors [35]. In other words, individuals feeling lonely are more inclined to use social media as a means of escaping real-life social interactions. The immediate feedback obtained during social media use can temporarily alleviate feelings of anxiety, leading individuals to engage in problematic social media use. Prolonged problematic use of social media can result in the loss of real-life social skills and social networks, in turn increasing feelings of loneliness.

Some studies suggest that college students who experience loneliness in the real world are more likely to expand their social networks through online social media [36]. However, college students with stronger feelings of loneliness tend to perform relatively poorly in online interpersonal interactions and online self-disclosure [17]. This not only fails to alleviate loneliness but may also lead to detachment from real-life interpersonal relationships, thereby further intensifying feelings of loneliness [17]. Excessive mobile internet use by college students, rather than alleviating their loneliness, may further disconnect them from real-world interpersonal relationships, consequently intensifying feelings of loneliness [17]. Other research suggests that lonely individuals often lack adequate social skills and may develop compulsive internet use behavior, which results in negative life outcomes rather than resolving their initial issues (e.g., problematic social media use). This intensified negative outcome further isolates individuals from healthy social activities, ultimately heightening their loneliness [16]. In summary, loneliness and problematic social media use mutually influence each other, creating a vicious cycle.

Most previous research has primarily relied on cross-sectional data, which generally indicates that loneliness can positively predict problematic social media use and vice versa, but the mutual predictive relationship between the two is still unclear. Moreover, although a few longitudinal studies have attempted to explore the mutual predictive relationship between loneliness and problematic social media use, these studies typically rely on data from only two time points, thus limiting their ability to accurately determine the mutual predictive relationship between the two. Therefore, this study aims to examine the mutual predictive relationship between loneliness and problematic social media use by employing a cross-lagged model with three time points. This approach not only provides a more in-depth perspective on the interaction between the two variables but also helps identify long-term trends and potential causal mechanisms.

### The developmental relation of loneliness and problematic social media use over time

College students are still in the adolescent stage of life development. Feelings of loneliness and problematic social media use are not static but rather exhibit developmental changes [20–23]. Research indicates that there are discernible trends in the development of both feelings of loneliness and problematic social media use. With regard to the developmental trend of loneliness, multiple studies have found that the levels of loneliness in adolescents in Western countries tend to decrease over time [37]. For instance, Ladd and Ettekal's [38] research with a sample of 478 American adolescents found that most adolescents feel lonelier in early adolescence than in late adolescence. Qualter's tracking study with 586 adolescents in England found that feelings of loneliness in adolescents generally stabilize and increase up to the age of 13, after which they rapidly decline [39]. Vanhalst et al.'s [40] five-wave longitudinal study of 389 Dutch adolescents aged 15–20 revealed that their feelings of loneliness typically decreased over time. Similarly, Lasgaard et al. [21] observed a declining trend in feelings of loneliness among Danish high school students. Mund et al. [20] conducted a meta-analysis of data from 75 longitudinal studies (*N* = 83,679) spanning Asia, Australia, Europe, and North America to examine the developmental trajectory of loneliness across the entire lifespan. Their results indicated that loneliness follows an inverted U-shaped trajectory over the course of a person's life and, on average, reaches its peak during the adolescent years [20]. These studies have predominantly been conducted in Western countries. There is a paucity of tracking studies on feelings of loneliness among Chinese college students, leaving a gap in evidence regarding the developmental levels of loneliness in this population.

On the other hand, with respect to the developmental trend of problematic social media use, while research results vary across different cultural backgrounds, the majority of studies support the notion that problematic social media use in adolescents generally increases over time. A two-year longitudinal tracking study involving Estonian female high school students showed a linear increase in their levels of problematic social media use over time [22]. However, other research has found that Dutch middle school students exhibited an initial increase in problematic social media use levels followed by a subsequent decrease over time [23]. Most of these studies have focused on adolescents in middle school, and there is a lack of research on problematic social media use in the college stage. Therefore, we conducted a longitudinal study of Chinese college students to provide additional evidence to accurately describe the developmental trajectory of problematic social media use among Chinese students.

In summary, previous research has described the developmental trajectories of loneliness and problematic social media use separately. Some studies have also provided static evidence supporting a significant positive correlation between feelings of loneliness and problematic social media use [11, 14–19]. However, these studies often fail to consider the changing relationship between these two variables over time and thus lack a dynamic perspective on the temporal development of the relationship between feelings of loneliness and problematic social media use. Therefore, this study employs latent growth models to explore the dynamic developmental patterns and influencing mechanisms of feelings of loneliness and problematic social media use.

### The present study

Previous cross-sectional studies failed to establish the mutual predictive relationship between feelings of loneliness and problematic social media use among college students. The limited longitudinal studies have often collected data at only two time points and lack investigations of the developmental trajectories of these variables and their potential dynamic interplay. Therefore, this study primarily focuses on two core research questions: (a) What is the causal direction of the relationship between feelings of loneliness and problematic social media use among college students as time progresses? (b) What are the developmental trajectories of feelings of loneliness and problematic social media use in college students over time, and how do they mutually influence each other? To address these questions, this study employs a cross-lagged approach to reveal the mutual predictive relationship between feelings of loneliness and problematic social media use among college students. Furthermore, it utilizes a latent growth model to elucidate the developmental trajectories of these variables and the dynamic mechanisms of their mutual influence over time. This research contributes to theoretically establishing the mutual predictive relationship. Some studies suggest that college students between feelings of loneliness and problematic social media use in college students and explaining their changes over time and their reciprocal interactions. Furthermore, this study can contribute to practical efforts to mitigate feelings of loneliness and problematic social media use among college students.

## Methods

### Participants and procedure

This study is based on a longitudinal investigation of college students' social media use. Utilizing cluster sampling, a total of 877 students from four universities in China were selected as the study participants. All participants completed surveys in September 2022 (Time 1), March 2023 (Time 2), and September 2023 (Time 3). During the second data collection, due to students either failing to provide their student ID numbers or reasons such as dropout and transfer that resulted in a dropout rate of 22.349%, 681 valid questionnaires were collected and analyzed. A t test was conducted to compare the first-time data of participants who dropped out in the second round with those who remained in the study and revealed no significant differences in feelings of loneliness and problematic social media use (*t* = 0.610, *p* > 0.05; *t* = -1.099, *p* > 0.05). Similarly, in the third data collection, 538 valid questionnaires were collected and analyzed, with a dropout rate of 20.999%. A t test was performed to compare the first-time data of participants who dropped out in the third round with those who remained in the study, and no significant differences were found in feelings of loneliness and problematic social media use (*t* = 0.299, *p* > 0.05;* t* = -1.643, *p* > 0.05). These findings indicate that there was no structured attrition in the study. Finally, the total valid sample size for participants who completed all three survey rounds was 538. Their average age was 18.24 ± 1.09 years, with males accounting for 36.4% and females accounting for 63.6%. Regarding their family's place of residence, 53% came from rural or town areas and 47% came from urban areas, with 12.3% serving as class cadres.

This study adhered to the principles of the research ethics committee of the institution and received approval from the principal of the participating school. Participants were informed of the purpose of the research, the voluntary nature of participation, and how to withdraw from the study. Informed consent was obtained from all participants before their involvement in the research. After each survey was completed, all participants received a small token of appreciation as compensation.

### Measures

Self-report assessments were used to collect the data. All items for loneliness and problematic social media use were measured on a 5-point Likert scale ranging from 1 = absolutely disagree to 5 = absolutely agree. All the negatively worded questions were reverse coded. Participants also reported their demographic information at the three waves of data collection.

### Loneliness measures

The three waves of loneliness among college students were assessed using the UCLA Loneliness Scale, version 3, developed by Russell [41]. This scale is a single-factor structure consisting of 20 items, including 11 positively scored items and 9 negatively scored items. An example item is, "How often do you feel that your interests and ideas are not shared by those around you?" For scoring, the values of negatively scored items were reversed before being summed with the positively scored items. A higher score indicated a greater sense of loneliness in students. In this study, the Cronbach's alpha coefficients for the scales were 0.915 at Time 1, 0.901 at Time 2, and 0.894 at Time 3.

### Problematic social media use measures 

The level of problematic social media usage among college students was measured using the Problematic Mobile Social Media Usage Assessment Scale for Adolescents, developed by Jiang [42]. This scale consists of five dimensions, increased stickiness, physical damage, fear of missing out, cognitive failure, and guilt, with a total of 20 items. An example is, "Unconsciously and frequently browsing mobile apps, checking friend updates on a daily basis, and not being able to recall how many times." All items in this scale were positively worded. The scores from all items were summed, and a higher score indicated a higher level of problematic social media usage among students. In this study, the Cronbach's alpha coefficients for the scales were 0.942 at Time 1, 0.962 at Time 2, and 0.972 at Time 3.

### Data analysis

First, this study used SPSS 26.0 and Mplus 8.3 for data processing. The descriptive statistics of the variables and their correlation coefficients were tested to examine the stability of loneliness and problematic social media use among college freshmen in China and their correlation at different time points.

**Fig. 1:**
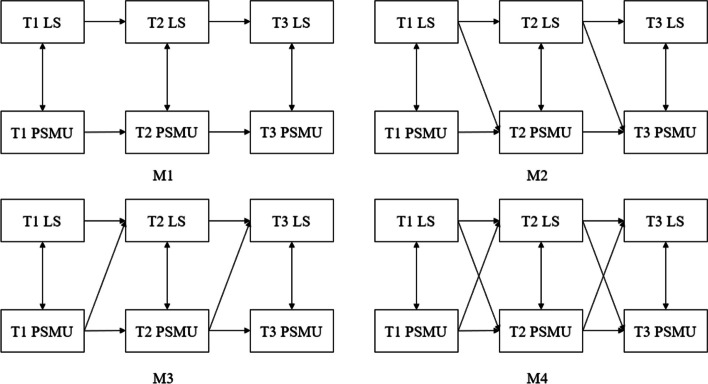
Nested models of the relationship between loneliness and problematic social media use. Note. LS = loneliness; PSMU = problematic social media use; T1, T2, T3 = Time 1, Time 2, Time 3

Second, we constructed a cross-lagged model of loneliness and problematic social media use to further examine the causal relationship between the two. For the cross-lagged model, we not only tested whether the hypothesized models showed a good fit to our data but also evaluated nested models (M1–M3). As shown in Fig. 1, Model 1 hypothesizes significant autoregressive relationships between loneliness and problematic social media use (the baseline model). However, Models 2 and 3 hypothesize a single-lagged relationship between loneliness and problematic social media use. Model 2 hypothesizes that loneliness at Time 1 and Time 2 influences problematic social media use across time (freely estimated), and the path coefficient from problematic social media use to loneliness is fixed to zero. Model 3 hypothesizes that problematic social media use at Time 1 and Time 2 influences loneliness across time (free estimated); the path coefficient from loneliness to problematic social media use is fixed to zero. Model 4 is a cross-lagged model containing all paths of Models 1, 2 and 3. Model 4 was compared with Models 1, 2 and 3 to determine the model that best fit the data.

Third, according to Muthén and Muthén's (2010) approach, the latent growth model with parallel processes involved two steps: (a) separately modeling the unconditional latent growth of loneliness and problematic social media usage across the three measurements and examining whether the development of loneliness and problematic social media usage exhibited linear growth and whether there were significant individual differences in the initial levels and growth rates of loneliness and problematic social media usage and (b) conducting a joint analysis of both processes. Based on the latent growth models of loneliness and problematic social media usage, a latent growth model with parallel processes was established to investigate the relationship between changes in loneliness and problematic social media usage.

We used Mplus [43] to conduct the analyses of model fit. All parameters were estimated using the full information maximum likelihood method. Model fit assessment used fit indices with acceptable critical values of χ2, df, CFI > 0.900, TLI > 0.950, RMSEA < 0.100, SRMR < 0.08 [44]. However, due to the sensitivity of χ2 to sample size, it is often not used as the sole criterion for assessing model fit [45].

## Results

### Common method bias

This study collected data through self-reported measures from students, which might introduce potential common method bias. Following the recommendations of Malhotra et al. [46] (2006) and Podsakoff et al. [47], we conducted Harman's single-factor test to assess common method bias separately for the three measurements. The results revealed that in all three tests, the number of factors with eigenvalues greater than 1 was equal to or greater than 2, and the variance explained by the first factor was 32.04%, 38.20%, and 42.34%, respectively. All these percentages were below the critical threshold of 50%, indicating the absence of substantial common method bias in this study.

### Descriptive statistics and correlation analysis

The means, standard deviations, and correlation matrices for college students' loneliness and problematic social media use at the three time points are presented in Table 1. The results show that from T1 to T3, loneliness was significantly positively correlated with problematic social media use (*rs* = 0.256–0.525, *ps* < 0.01). Loneliness at each time point was also positively correlated (*rs* = 0.567–0.738, *ps* < 0.01), as was problematic social media use (*rs* = 0.393–0.599, *ps* < 0.01). This indicates that the concurrent correlations and stability of loneliness and problematic social media use in college students were consistent, making them suitable for cross-lagged model analysis and latent growth modeling.

**Table 1:** Means, standard deviations, and correlations among main measures

Variable	M(SD)	1	2	3	4	5	6
1.T1 LS	2.460(0.580)	1.000					
2.T2 LS	2.540(0.559)	0.674**	1.000				
3.T3 LS	2.631(0.551)	0.567**	0.738**	1.000			
4.T1 PSMU	2.556(0.698)	0.424**	0.343**	0.322**	1.000		
5.T2 PSMU	2.661(0.733)	0.283**	0.441**	0.426**	0.501**	1.000	
6.T3 PSMU	2.691(0.716)	0.256**	0.396**	0.525**	0.393**	0.599**	1.000

*Note*. *LS* loneliness, *PSMU* problematic social media use, *T1, T2, T3* Time 1, Time 2, Time 3

^**^p < 0.01

### Cross-lagged model analysis

To examine the causal relationship between college students' loneliness and problematic social media use, this study conducted a cross-lagged analysis of the three-wave measurements of loneliness and problematic social media use following the recommendations of Martens and Haase [48]. Before investigating the relationships between variables in the cross-lagged analysis, as shown in Fig. 1, the fit indices of the four models were compared. Table 2 presents the fit indices for the four models in the current study and the results of chi-square differences between each competing model (M1, M2, and M3) and the full model (M4).

**Table 2:** The goodness-of-fit statistics for the nested models

model	χ^2^	df	CFI	TLI	SRMR	RMSEA	model comparisons	Δχ^2^	Δdf	*p*
M1	70.820	8	0.953	0.917	0.106	0.121	M4 vs.M1	49.109	4	< 0.001
M2	40.630	6	0.974	0.939	0.053	0.104	M4 vs.M2	18.919	2	< 0.001
M3	44.628	6	0.971	0.932	0.072	0.109	M4 vs.M3	22.917	2	< 0.001
M4	21.711	4	0.987	0.953	0.022	0.091				

^*^p < 0.05

^**^p < 0.01

^***^p < 0.001

From the fit indices in Table 2, it is evident that M4 outperformed M1, M2, and M3, with significant chi-square differences between M4 and M1 (Δχ^2^ = 49.109, Δdf = 4, *p* < 0.001), M4 and M2 (Δχ^2^ = 18.919, Δdf = 2,* p* < 0.001), and M4 and M3 (Δχ^2^ = 22.917, Δdf = 2,* p* < 0.001). These results suggest that M4 is the optimal model among the four competing models mentioned above.

**Fig. 2:**
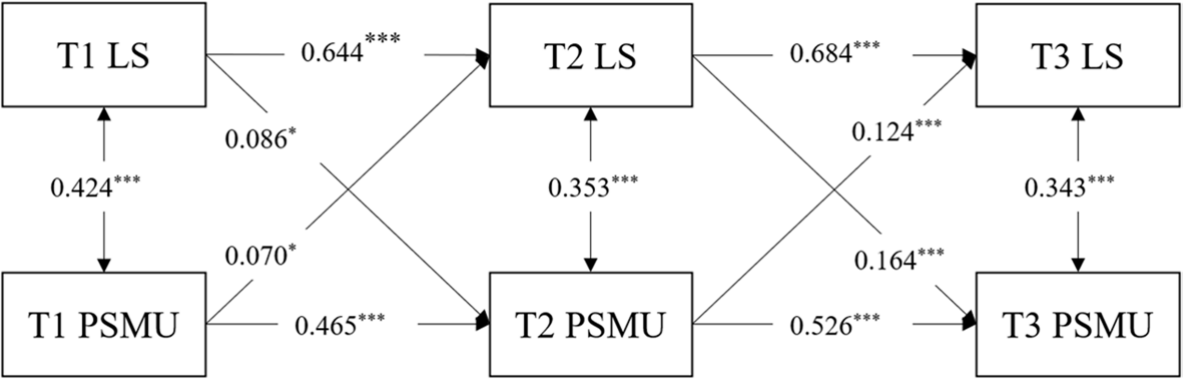
Cross-lagged model of loneliness and problematic social media use. Note. LS = loneliness; PSMU = problematic social media use; T1, T2, T3 = Time 1, Time 2, Time 3. ^*^ *p* < 0.05; ^**^ *p* < 0.01; ^***^ *p* < 0.001

The final model for college students' loneliness and problematic social media use is depicted in Fig. 2. This model exhibits a good fit (χ^2^/df = 5.428, CFI = 0.987, TLI = 0.953, SRMR = 0.022, RMSEA = 0.091). As observed in Fig. 2, from T1 to T3, loneliness and problematic social media use display considerable stability, with standardized autoregressive path coefficients ranging from 0.644 to 0.684 (*p* < 0.001) and 0.465 to 0.526 (*p* < 0.001), respectively. In terms of cross-predictive paths, T1 loneliness significantly predicts T2 problematic social media use (β = 0.086, *p* < 0.05), and T1 problematic social media use significantly predicts T2 loneliness (β = 0.070, *p* < 0.05). Similarly, T2 loneliness significantly predicts T3 problematic social media use (β = 0.164, *p* < 0.001), and T2 problematic social media use significantly predicts T3 loneliness (β = 0.124, *p* < 0.001).

In summary, the results of the cross-lagged model analysis consistently support the mutual predictive relationship between college students' loneliness and problematic social media use. These findings further underscore that both loneliness and problematic social media use among college students are relatively stable outcomes.

### Development trajectories of college students' loneliness and problematic social media use

#### Unconditional latent growth model for college students' loneliness

**Fig. 3:**
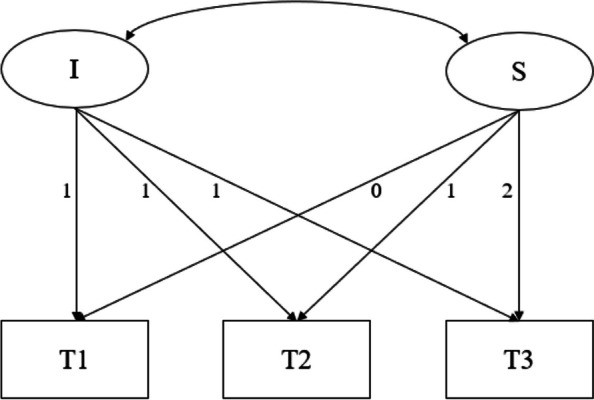
LGM with unconditional variables of loneliness and problematic social media use. Note. LGM = latent growth model; I = intercept; S = slope; T1, T2, T3 = Time 1, Time 2, Time 3

To examine the changing trend in college students' loneliness, an unconditional latent variable linear growth model was constructed (see Fig. 3). The intercept represents the initial value of loneliness at the first measurement with all factor loadings fixed at 1. The slope represents the linear change rate of loneliness with factor loadings set at 1, 2, and 3 due to the equal time intervals between the three measurements [49]. In this way, the intercept of the model represents the initial level of loneliness for participants upon entering college, while the slope represents the change in loneliness at each measurement point. The results reveal that the unconditional linear model provides a good fit for the data (χ^2^/df = 1.340, CFI = 1.000, TLI = 1.000, RMSEA = 0.000, SRMR = 0.003).

In the unconditional latent variable linear growth model for loneliness, the intercept, representing the initial level of loneliness, is 2.459 (SE = 0.024, *p* < 0.001), which is significantly greater than 0. The slope, indicating the linear upward trend of loneliness over the three measurements, is 0.086 (SE = 0.011, *p* < 0.001). Furthermore, the estimated variances of the intercept factor (σ^2^ = 0.255, SE = 0.023, *p* < 0.001) and the slope factor (σ^2^ = 0.041, SE = 0.009, *p* < 0.001) are both significantly different from 0.001, indicating significant individual differences in both the initial level of loneliness and the rate of increase in loneliness over time. Finally, there is a significant negative correlation between the intercept growth factor and the slope growth factor (*r* = -0.360, *p* < 0.001), suggesting that students with lower initial levels of loneliness experience a faster increase in loneliness over the three measurements (see Table 3).

**Table 3:** Coefficient and fit indices of unconditional latent growth model of SE and CT

Model	Fit indices					Coefficients		Variance	
	χ^2^/df	CFI	TFI	SRMR	RMSEA	Intercept	Slope	Intercept	Slope
LS	1.340	1.000	1.000	0.003	0.000	2.459^***^	0.086^***^	0.255^***^	0.041^***^
PSMU	2.441	0.996	0.989	0.016	0.052	2.567^***^	0.065^***^	0.315^***^	0.088^***^

*Note*. *CT*, loneliness, *PSMU* problematic social media use, *T1, T2, T3* Time 1, Time 2, Time 3

^*^p < 0.05

^**^p < 0.01

^***^p < 0.001

#### Unconditional latent variable linear growth model for problematic social media usage

To examine the changing trend of problematic social media usage among college students, an unconditional latent variable linear growth model is constructed (see Fig. 3). The model structure and significance are similar to the unconditional latent variable linear growth model for loneliness. The unconditional linear model fit the data well (χ^2^/df = 2.441, CFI = 0.996, TLI = 0.989, RMSEA = 0.052, SRMR = 0.016). In the unconditional latent variable linear growth model for problematic social media usage, the model's intercept, representing the initial level of problematic social media usage, is 2.567 (SE = 0.029, *p* < 0.001), which is significantly greater than 0. The model's slope, representing the linear increase in problematic social media usage over the three measurement periods, is 0.065 (SE = 0.017, *p* < 0.001). Furthermore, the variance estimates for the intercept factor (σ^2^ = 0.315, SE = 0.038, *p* < 0.001) and the slope factor (σ^2^ = 0.088, SE = 0.017,* p* < 0.001) are both significant at the 0.001 level. This indicates that there are significant individual differences in both the initial level of problematic social media usage and the rate of change in problematic social media usage over time.

Finally, there is a significant negative correlation between the intercept growth factor and the slope growth factor (*r* = -0.357, *p* < 0.001), suggesting that students with lower initial levels of problematic social media usage exhibit faster increases or decreases in problematic social media usage over the three measurement periods (see Table 3).

### Parallel development latent growth model for loneliness and problematic social media usage

In the previous sections, we explored the mutual predictive relationship between loneliness and problematic social media usage using cross-lagged models. To further examine the dynamic interplay between these two variables, we constructed a parallel development latent growth model to simultaneously investigate the latent growth in loneliness and problematic social media usage among college students.

**Fig. 4:**
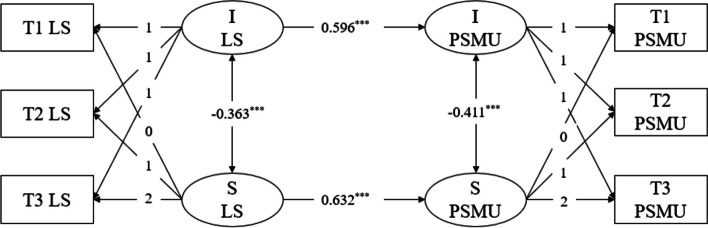
LGM of loneliness and problematic social media use. Note. LGM = latent growth model; I LS = intercept of loneliness; S LS = slope of problematic social media use; I PSMU = intercept of problematic social media use; S PSMU = slope of problematic social media use; T1, T2, T3 = Time 1, Time 2, Time 3

First, we used the intercept and slope from the loneliness model to predict the linear growth in problematic social media usage. The model fit the data well (χ^2^ (7) = 4.305, CFI = 0.984, TLI = 0.966, RMSEA = 0.078, SRMR = 0.018), allowing us to proceed with further analysis. The regression results of the parallel development latent growth model for loneliness and problematic social media usage are presented in Fig. 4. The slope of loneliness positively predicted the slope of problematic social media usage (β = 0.596, SE = 0.051, *p* < 0.001), indicating that as loneliness increases more rapidly over time, students' problematic social media usage levels also increase more rapidly. The intercept of loneliness positively predicted the intercept of problematic social media usage (β = 0.632, SE = 0.098, *p* < 0.001), suggesting that students with higher initial levels of loneliness exhibit higher initial levels of problematic social media usage. Furthermore, there was a negative correlation between the intercept growth factor and the slope growth factor of loneliness (*r* = -0.363, *p* < 0.001), indicating a negative relationship between the initial level and growth rate of loneliness. Specifically, students with lower initial levels of loneliness experience a faster increase in loneliness. Similarly, there was a negative correlation between the intercept growth factor and the slope growth factor of problematic social media usage (*r* = -0.411, *p* < 0.001), suggesting a negative relationship between the initial level and growth rate of problematic social media usage. Students with lower initial levels of problematic social media usage experience a faster increase in usage.

**Fig. 5:**
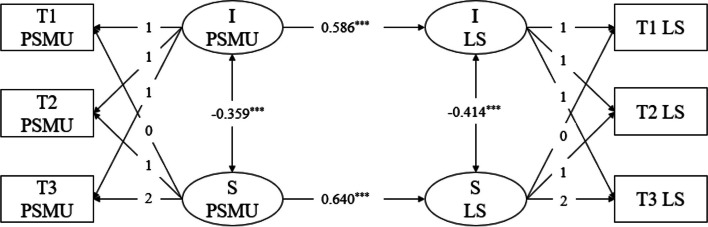
LGM of problematic social media use and loneliness. Note. LGM = latent growth model; I PSMU = intercept of problematic social media use; S PSMU = slope of problematic social media use; I LS = intercept of loneliness; S LS = slope of problematic social media use; T1, T2, T3 = Time 1, Time 2, Time 3

Next, we used the intercept and slope from the problematic social media usage model to predict the linear growth of loneliness. This model demonstrated good fit (χ^2^ (7) = 4.305, CFI = 0.984, TLI = 0.966, RMSEA = 0.078, SRMR = 0.018), making it suitable for further analysis. In the parallel development latent growth model of problematic social media usage and loneliness, the regression results, as shown in Fig. 5, revealed that the slope of problematic social media usage significantly predicted the slope of loneliness (β = 0.586, SE = 0.047, *p* < 0.001). This suggests that as time progresses, a faster increase in problematic social media usage is associated with a faster increase in students' levels of loneliness. Additionally, the intercept of problematic social media usage positively predicted the intercept of loneliness (β = 0.640, SE = 0.095,* p* < 0.001), indicating that higher initial levels of problematic social media usage were associated with higher initial levels of loneliness. Furthermore, a negative correlation was observed between the intercept growth factors and the slope growth factors. The correlation coefficient between the intercept growth factor of problematic social media usage and the slope growth factor of loneliness was -0.363 (*p* < 0.001), indicating that higher initial levels of problematic social media usage were related to faster increases in loneliness. Similarly, the correlation coefficient between the intercept growth factor of loneliness and the slope growth factor of problematic social media usage was -0.414 (*p* < 0.001), indicating that lower initial levels of loneliness were associated with faster increases in problematic social media usage.

## Discussion

### Bidirectional predictive relationship between loneliness and problematic social media use in chinese college students

This study utilized a cross-lagged model to explore the influence between loneliness and problematic social media use in Chinese college students. The results of the study showed that in the three waves of testing, loneliness positively predicted problematic social media use and problematic social media use positively predicted loneliness, indicating a mutual positive influence between the two. This result does not negate the first and second viewpoints presented earlier; instead, it integrates them and supports the third perspective, which suggests that heightened loneliness exacerbates problematic social media use in Chinese college students and, in turn, further intensifies their feelings of loneliness.

The results of this study indicate a bidirectional predictive relationship between loneliness and problematic social media use among Chinese university students. Specifically, lonely individuals often perceive it as easier to establish and maintain interpersonal relationships in social networks compared to real-life situations [27]. Behind the screen of social media platforms, individuals are protected by the anonymity of the internet, making it easier for them to open up and communicate with others online. Social media expands the social horizons of lonely individuals and compensates for their social deficits in real life [11]. It provides them with a sense of belonging, which leads to their immersion in social media and the emergence of problematic social media usage. Although social media usage (i.e., interacting while physically isolated) may appear to temporarily alleviate an individual's loneliness, interactions on social media primarily involve simple text responses and written comments. In comparison to face-to-face offline interactions, online social media interactions are superficial and indirect, leading to a sense of technological alienation among individuals [33]. Depending on such superficial and indirect interactions to bridge the psychological distance between individuals for an extended period can reduce the depth and quality of interactions with family and friends, which negatively affects the relationships of university students with their family and friends [50, 51]. Furthermore, when individuals engage in extensive and long-term use of social media, they may unintentionally replace real-world interactions with virtual relationships, increasing their perception of social isolation [18]. Consequently, this exacerbates their feelings of loneliness.

This finding in our study offers insights for promoting the psychological well-being of college students and intervening in problematic social media use. First, implementing measures to either reduce loneliness or decrease problematic social media use can help break the cycle of mutual reinforcement between loneliness and problematic social media use in college students. Second, taking a comprehensive approach by addressing both loneliness reduction and problematic social media use reduction interventions may be more effective because they can work synergistically, disrupting the vicious cycle between these two issues and contributing to improvements in psychological well-being and social media usage habits.

### Development of loneliness in chinese college students

This study found an increasing trend of loneliness among Chinese college students during their academic tenure, aligning closely with Erikson's theory of eight stages of life development. Erikson posits that the primary task during the ages of 18–25 is to establish intimacy and avoid feelings of loneliness. In the university stage, students face the challenge of establishing new social networks while attempting to strike a balance between forming intimate relationships with peers and maintaining independence from family and friends. The instability of these social networks and the adaptation to independent living may exacerbate their feelings of loneliness [52]. Furthermore, during the COVID-19 pandemic, many university students sought relief from isolation-induced loneliness through social media. However, with the easing of the pandemic and the gradual resumption of campus life, factors such as academic pressure and difficulties adapting to campus life have significantly increased psychological issues among university students (such as anxiety, depression, and smartphone addiction) [53]. These psychological problems further contribute to the interpersonal isolation of university students, intensifying their feelings of loneliness [21].

These findings reflect the unique challenges faced by Chinese university students within a distinctive sociocultural context. Under the combined influence of various factors, the sense of loneliness among university students in China is progressively on the rise. These results offer a crucial perspective for understanding the psychological well-being of Chinese university students.

### Development of problematic social media usage among chinese college students

This study found that the level of problematic social media usage among Chinese college students increases continuously during their college years, which is consistent with previous research findings [22]. First, the development of internet technology and the widespread use of mobile devices provide fertile ground for problematic social media usage. China is one of the world's largest internet markets, with a very high penetration rate of internet and smartphones. With the advancement of internet technology, social media platforms have become more accessible and user-friendly. These platforms offer a wide variety of content and interactions that attract students to spend more time online, making them more susceptible to social media addiction. Second, social media platforms provide various forms of entertainment content, including videos, music, and games. These types of content can capture the interest of college students, leading them to engage with these platforms for entertainment. However, at times, they may become engrossed and find it challenging to disengage. Finally, social media can lead to social comparison and anxiety and can contribute to problematic social media usage. College students may see the lives and accomplishments of others on social media, which may lead to self-comparisons and feelings of unease [54]. This may drive them to spend more time online seeking social approval or escaping from real-life issues [55]. With the combined influence of these factors, the level of problematic social media usage among Chinese college students has been on the rise.

### Mutual developmental impact of loneliness and problematic social media usage in chinese university students

The results of this study indicate that the slope of loneliness in Chinese university students positively predicts the slope of problematic social media usage, and the slope of problematic social media usage positively predicts the slope of loneliness. In other words, as time progresses, the faster loneliness increases, the faster the level of problematic social media usage in students increases; in turn, the faster problematic social media usage increases, the faster students' levels of loneliness rise.

There may be three possible explanations for this complex relationship. Firstly, the emotion regulation mechanism may be crucial in explaining this association. When college students experience loneliness in real life, they may turn to online social media for interaction, recognition, and emotional comfort [56]. However, this approach can only partially fulfill the emotional needs of college students and exacerbate compulsive social media usage behaviors. Additionally, inevitable upward social comparison and negative interpersonal evaluations in online social interactions can increase psychological distress and feelings of loneliness [56]. During periods of emotional distress, college students may find themselves caught in the contradiction of relying on social media to regulate emotions while fearing negative interpersonal evaluations. This not only increases problematic social media usage among college students but also leads to a lack of deep emotional communication and effective emotional support in online social media interactions, thereby intensifying the development of loneliness. This establishes a trend of gradual enhancement and mutual influence between the two over time.

The I-PACE model (Interaction of Person-Affect-Cognition-Execution) provides a robust framework for understanding the interaction between loneliness and problematic social media use. This model emphasizes the dynamic interactions among individual traits (P), emotional states (A), cognitive processes (C), and addictive behaviors (E) (I) [35]. Specifically, when individuals (P) experience loneliness (A), they seek satisfaction for their psychological needs through engaging with social media (E). The sense of satisfaction provided by social media usage prompts individuals to increase the frequency of use, leading to problematic social media usage behaviors (E). Over time, this dependency on social media may result in a decline in individuals' social skills in real life (E), consequently enhancing feelings of loneliness (A), creating a self-reinforcing negative cycle (I) [57]. This outcome suggests a mutual predictive relationship between loneliness and problematic social media use.

Once again, based on the "Risk Factors—Risk Factors Model," we can comprehend the mutually reinforcing effects of loneliness and Problematic Social Media Use (PSMU) over time. In this model, loneliness serves as a risk factor, prompting individuals to engage in excessive use of social media as a coping mechanism. As time progresses, the overreliance on virtual social interactions may exacerbate loneliness due to a lack of profound and meaningful face-to-face interactions. Consequently, a vicious cycle of mutual reinforcement forms between loneliness and PSMU, intensifying their destructive impact over time [58].

Furthermore, this study found that the intercept of loneliness positively predicts the intercept of problematic social media use (PSMU), and the intercept of problematic social media use positively predicts the intercept of loneliness. In other words, the initial level of loneliness positively predicts the initial level of problematic social media use, and vice versa. This contradicts the conclusion of existing research that problematic social media use does not harm mental health [59]. A possible explanation is that the interaction mechanism between the social media usage habits of Chinese university students and loneliness is unique. Specifically, social media has become the primary channel for adolescents in modern society to engage in interpersonal interactions [60]. Many Chinese university students have been exposed to social media since childhood, and some students may exhibit problematic social media use behavior even before entering university. Additionally, under the high-pressure examination system, academic performance is the sole measure of student success [61]. By reducing the time spent on interpersonal interactions, many students invest more time in studying to achieve good results in the college entrance examination. This approach leads to loneliness in many Chinese students academically, even causing anxiety and depression, subsequently immersing themselves in social media usage to alleviate academic pressure and escape the loneliness in reality [62]. Furthermore, individuals' online usage habits do not undergo significant changes in a short period, and there is a significant correlation between online behavior and loneliness [17]. The resurgence of the COVID-19 pandemic in 2022 forced most university students to shift their lives and studies to online mode. In this situation, social media became their primary tool for socializing, but this mode may lead to problematic social media use behavior in students. Even in the restored normal campus life, students immersed in social media usage may find themselves lonelier in the real world, as they cannot establish real and high-quality interpersonal interactions with peers in a short time. In summary, there is a mutual influence between the initial levels of problematic social media use and loneliness. This result provides a new perspective for understanding the relationship between loneliness and social media usage behavior in university students and offers important insights for future psychological interventions and educational strategies.

## Limitations and future directions

While this study yields significant findings, it is not without limitations. First, both loneliness and problematic social media use are complex psychological and behavioral conditions that have been widely investigated in previous research [63–65]. Relevant factors may include personality, psychological, social, and cultural elements. In comparison to the multitude of influencing factors discovered in prior studies, this research was relatively narrow in scope and focused solely on the relationship between loneliness and problematic social media use. Consequently, this study may overlook the influence of other variables, such as personality, age, gender, academic grade information. Future research should endeavor to develop more intricate theoretical models to address this issue, such as more finely distinguishing between students with different academic performances in terms of loneliness and problematic social media use behavior. Second, the longitudinal tracking of the sample in this study was limited. Chinese university degree programs typically span 3–4 years, with some professional programs extending to 5 years. This study tracked data for only one year and thus presented results for a one-year developmental period rather than the trajectory of change during a student's entire college period. Future research should begin tracking data from the first year of college and continue to graduation, allowing for a comprehensive investigation of the developmental trajectories and mutual influences of loneliness and problematic social media use during a student's entire college experience. Third, the data collected in this study rely heavily on self-report measures, which are only indirect indicators of college students' psychological and behavioral aspects. Therefore, caution should be exercised in interpreting these results. In future research, it is advisable to supplement self-reported data with additional data sources, such as student interviews and observations, to address the limitations associated with relying solely on self-reported data.

## Conclusions

This study utilized cross-lagged regression analysis and latent growth modeling to explore the causal and developmental relationships between loneliness and problematic social media use among Chinese university students. Our research findings indicate the following: (a) loneliness and problematic social media use mutually and positively influence each other to establish a reciprocal causal relationship; (b) during their university years, Chinese students experience gradual increases in both loneliness and problematic social media use levels; (c) the intercept and slope of loneliness significantly positively influence the intercept and slope of problematic social media use, and conversely, the intercept and slope of problematic social media use significantly positively influence the intercept and slope of loneliness. These discoveries reveal the longitudinal dynamics between loneliness and problematic social media use among Chinese university students. These results provide valuable insights for researchers and educators to intervene in the development of loneliness and problematic social media use in university students. Interventions targeting either aspect can contribute to reducing the levels of both.

## Data Availability

The data that support the findings of this study are available on request from the corresponding author Feng. The data are not publicly available because they contain information that could compromise the privacy of the research participants.
